# Predicting Consumer Green Product Purchase Attitudes and Behavioral Intention During COVID-19 Pandemic

**DOI:** 10.3389/fpsyg.2021.760051

**Published:** 2022-01-25

**Authors:** Xia Chen, Muhammad Khalilur Rahman, Md. Sohel Rana, Md. Abu Issa Gazi, Md. Atikur Rahaman, Noorshella Che Nawi

**Affiliations:** ^1^School of Management, Jiujiang University, Jiujiang, China; ^2^Faculty of Entrepreneurship and Business, Universiti Malaysia Kelantan, Pengkalan Chepa, Malaysia; ^3^Faculty of Business and Accountancy, University of Malaya, Kuala Lumpur, Malaysia; ^4^School of E-Commerce, Jiujiang University, Jiujiang, China; ^5^Global Entrepreneurship Research Innovation Centre (GERIC), Universiti Malaysia Kelantan, Pengkalan Chepa, Malaysia

**Keywords:** green product, consumers’ attitudes, COVID-19 pandemic, green product orientation, behavioral intention

## Abstract

This work has aimed to investigate the consumers’ green product purchase attitudes and behavioral intention during COVID-19 pandemic. Data was collected through a survey method of 503 consumers in Malaysia. Data were analyzed using the partial least square method. The findings revealed that fear of COVID-19 pandemic has a significant impact on green product behavioral intention. Green product literacy, green product orientation, and social influence have a significant influence on green product purchase attitudes. The results also indicated that consumers’ green product purchase attitudes mediate the effect of green product literacy, green product orientation, and social influence on behavioral intention. The findings of this work will provide strategically relevant references to green marketers and retail managers in the understanding of consumers’ green product purchase attitudes and green product behavioral intention during the ongoing uncertainty of the COVID-19 pandemic.

## Introduction

A safe and sustainable environment and economy have become a global commitment in recent years. The rapid industrial revolution has forced many scholars, economists, and professionals to visit industrial production processes, policies, and regulations to comply with environment-friendly approaches. The integration of green product consumption behavioral intention has led to a new environment of health consciousness due to the fear and ongoing uncertainty of the COVID-19 pandemic. The green concept has been taken eminent place from the production to the consumption of commodities by the end-users (Mishal et al., 2017; Ferraz et al., 2021). The industries are putting a great effort to remain sustainable in the global competitive open economy. Many companies are inefficiently over utilizing natural resources to produce consumer goods and services that cause harm to the environment by generating pollution in different forms (Shen et al., 2021).

The rapid industrialization process is not only harming environmental elements around us, but is also causing an immense health hazard to human health (Junsheng et al., 2020). Integrating green conception has become a great concern for all involved stakeholders in the production process, including the buyers of the consumer commodities. The behavioral intention of green products has been inspiring consumers due to its strong connections with environmental ethics and to mitigate adverse impacts on the global environment, particularly due to the fear of COVID-19 pandemic crisis; however, there is a gap between green product purchase attitudes and behavioral intention due to the outbreak of COVID-19 pandemic. The environmental literacy by the consumers has led the producers to rethink and redesign their products and services considering environmental issues in their production policies. The global concern of COVID-19 pandemic affects consumers’ green product purchasing attitudes and their green product consumption behavioral intention. In this work, we investigated the predicting factors that affected consumers’ green product purchasing attitudes and behavioral intention. This study focused on the fear of COVID-19 pandemic that affects consumers’ green product behavioral intention. In addition, we investigated the impact of green product literacy, green product orientation, and social influence on consumers’ green product purchasing attitudes and green product behavioral intention.

The adverse COVID-19 impacts fall on human lives through the fear of pandemics worldwide. The global leaders from around the world have taken a firm stance and discovered the vaccine against the outbreak of COVID-19 pandemic, which facilitates enhancing environmental values among the people and society. Peoples’ intention to purchase green or environment-friendly products (Kumar et al., 2021) has increased due to the fear of COVID-19 epidemic and health concerns. The green products purchase attitude and consumer behavioral intention have been a noble phenomenon to protect the COVID-19 environment and to save human health. Green purchase intention has been covered by many previous studies from time to time, for example, consumers’ green perceptions, green product purchase attitudes and sustainable green product behavioral intention, green labeling, and green supply chain management (Newaz et al., 2020; Shukor et al., 2020; Sun et al., 2021).

The demand for green products is significantly increasing in the global market and expanding effectively in developed nations. The green product purchase attitude has also hit significantly in developing nations like Malaysia. It is crucial to understand the consumers’ attitudes to purchasing green products for their behavioral intention (Yadav and Pathak, 2017). This study investigates the predictors of consumers’ attitudes to purchasing green products and behavioral intention during the COVID-19 pandemic.

## Literature Review

### Underpinning Theory

This work uses the theoretical lens of predictors of customers’ attitudes and their behavioral intention toward green products. It also adopts the concept of the theory of fear (Sun et al., 2021) to evaluate the consumer’s psychological factors related to fear of COVID-19 pandemic, green product literacy, green product orientation, and social influence. The concept of the theory of planned behavior (TPB) (Ajzen, 1991) is used in this study to focus on the consumers’ purchase attitudes and behavioral intention of green products for their healthy life due to the uncertainty of COVID-19 pandemic. Consumers’ psychological aspects indicate that fear is a natural and biological emotion of all humans. It involves a high individual emotional response and alerts the presence of thereat or danger of harm physically or psychologically. Based on the concept of the theory of fear (Sun et al., 2021) and TPB (Ajzen, 1991), this work conceptualizes the consumers’ psychological factors such as fear of COVID-19 pandemic, green product literacy, green product orientation, and social influence, which may affect consumers’ purchasing attitudes toward green product consumption behavioral intention. Myung et al. (2020) stated that the TPB dominated a number of studies related to green product consumption behavioral intention. In the context of green product behavioral intention, the earlier studies (Halder et al., 2020; Wang et al., 2020; Sun et al., 2021) applied TPB as a significant theoretical basis to realize consumers’ attitudes toward green product behavioral intention. This work investigates the impact of fear of COVID-19 pandemic, green product literacy, green product orientation, and social influence on consumers’ green product attitudes, which in turn reflect the consumers’ green product consumption behavioral intention during the COVID-19 crisis.

### Fear of COVID-19 Pandemic

The fear is a persuasive message designed to interact to scare individuals by resenting terrible outcomes of neglecting a specific caution. The spread COVID-19 pandemic can be segmented into fear control. Marketers use the fear technique to persuade consumers to purchase products (McDaniel and Zeithaml, 1984, and green product behavioral intention. Fear control guides emotional responses caused by risk. There are no apparent significant signs of controlling the spread of the COVID-19 epidemic. Fear of COVID-19 pandemic is an adaptive response to danger (Addo et al., 2020; Naeem, 2021). Due to the uncertainty of the ongoing pandemic of COVID-19, fear can become chronic and burdensome. Fear of COVID-19 can be a significant influence factor that reflects customers’ purchase decision of green products and behavioral intention. Addo et al. (2020) identified that fear of COVID-19 pandemic appeals to consumers favoring purchase behavior toward personal protective equipment. Sun et al. (2021) identified that the awe of COVID-19 pandemic affects consumers’ green product consumption behavioral intention. Jian et al. (2020) investigated the relationship between fear and uncertainty of the COVID-19 epidemic and green consumer behavior in the context of China. This study investigates the correlation between fear of COVID-19 pandemic and Malaysian consumers’ green product consumption behavioral intention. Thus, this study postulated that:

H1: Fear of COVID-19 pandemic has a significant impact on consumers’ green product consumption behavioral intention.

### Green Product Literacy

Green product literacy encompasses experiences, knowledge, and understanding of a wide range of green or environmental-friendly product concepts, facts, and issues. Green product literacy refers to an individual’s general ideas of facts and concepts of natural environmental-friendly products and the interdependent ecosystem. Green product knowledge or literacy can help to develop the foundation of belief about a particular issue related to green products and services (Kim and Stepchenkova, 2019). General people can gain knowledge related to environment-related issues through proper training, workshops, educational programs, or real-life experience. Biswas (2020) stated that green product literacy could reflect consumers’ attitudes to purchasing green products. The previous studies identify the association between knowledge and consumers’ attitudes toward green product purchasing behavior (Liobikienė and Poškus, 2019). Joshi and Rahman (2015) believed that green product literacy could lead to green product purchasing attitudes. However, some of the works have found an insignificant relationship between environmental knowledge and their behavioral intention to purchase green products. There is a lack of empirical studies’ focus on the green product literacy and green product attitudes toward consumers’ green product behavioral intention. Green product education will influence a certain level of ethical values regarding the environment of individuals by which the different manufacturing companies can create and maintain the green product for customer health security and safety. Thus, we propose that:

H2: Green product literacy has a positive impact on consumers’ green product purchasing attitudes.

### Green Product Orientation

The green product orientation means someone’s profound feelings for natural or eco-friendly products that are not harmful to health. When an individual is connected to natural environmental-friendly products, that individual is supposed to be nature-oriented. Consumers’ green product purchase attitudes can be influenced by consumers’ green product orientation (Sony and Ferguson, 2017). The personal attitude of green product consumption is acknowledged by green product orientation (Paswan et al., 2017; Elias, 2020). Amin et al. (2020) identified a significant relationship between green product orientation and proenvironmental behavior. Yu and Huo (2019) found a positive relationship between green product orientation and green management attitude. Danso et al. (2019) and Wickramasinghe (2019) found that green product orientation has a significant impact on consumers’ attitudes toward purchasing green products. Santra et al. (2021) explained the green product uniqueness orientation and export entrepreneurship. Moreover, when an individual feels that he is interconnected with the natural world, he will grow a self-sense that may include all other nonhuman living beings that will lead to biospheric concerns (Lee et al., 2021). In addition to this, humans need to respect the proper rights of the natural environment, green product orientation and its elements like plants, and animals should not destroy them inconsiderately. Therefore, love and the protective attitudes of the green environment will work as a crucial predictor of green product attitudes toward consumption behavioral intention. This study suggests the following hypothesis:

H3: Green product orientation has a positive impact on consumers’ attitudes to purchasing green products.

### Social Influence

Society is greatly influenced by traditional and cultural trends of individuals, groups, mass, and commercial media. Social influence refers to individuals changing their attitudes to meet the demands of product consumption behavioral intention. Social influence has many forms and it can be seen in conformity, peer pressure, obedience, sales, and marketing, which may lead to establishing the green product attitudes toward consumption behavioral intention of people. In this work, we investigated the relationship between the social influence of people’s green product purchasing attitudes and green product consumption behavioral intention for their healthy life and safety, particularly during the pandemic. Consumers’ social influence and green product purchase attitudes have been integrated into green consumer study. Wang (2014) indicated that social influence could play a crucial role in consumer decisions toward purchasing green products behavioral intention. Social influence on individuals’ behavior can be found in the social psychological theories (An et al., 2021; Mancini, 2021) and TPB by conceptualizing subjective norms. Social influence often evolves as the perceived social pressure that influences an individual’s attitudes (Ajzen, 1991). Ojo and Fauzi (2020) identified that social influence has a direct influence on a person’s attitudes. Some previous studies have also found a positive effect of social influence on individuals’ attitudes (Chen et al., 2021; Yılmaz and Anasori, 2021). Nugraha and Widyaningsih (2021) have identified that individuals’ attitudes are influenced mostly by the expectation of the consumer. A recent study has come up with a similar result acknowledging that consumers’ attitudes are influenced by their social group’s perceptions of products (Koo and Chung, 2014; Bratu, 2019). Social influence has many forms that may lead to establishing the attitude of green product consumption behavioral intention of people. Therefore, we postulate that:

H4: Social influence has a positive influence on consumers’ green product purchase attitudes.

### Green Product Purchase Attitude

Attitude refers to the degree to which an individual has a judgment of products and services (Ajzen, 1991). Previous studies have explained that attitude is a crucial antecedent of consumption behavioral intention (Graessley et al., 2019; Dhir et al., 2021). Fishbein et al. (1980) reported that the consumers’ strong positive attitude leads to consumption behavioral intention of products. Han et al. (2010) believed that people’s positive attitude strengthens their performance of consumption behavioral intention of certain products. Nguyen et al. (2019) found that consumers’ attitude is positively associated with green purchase behavior. People believed that green consumption behavioral intention might protect their health. Dhir et al. (2021) indicated that consumer’s attitudes could influence the consumption behavioral intention of green products but there is a recognized gap between attitudes and behavior. Shimul et al. (2021) reported that consumers’ environmental and natural green product-related knowledge and information could lead to an increase in consumer positive attitudes and purchase of green products behavioral intention. Dabija et al. (2018) and Ogiemwonyi and Harun (2020) indicated that a green attitude has a higher significant influence on green product consumption behavior. Consumers’ attitudes is the crucial predictor of consumer green product consumption behavioral intention (Han and Kim, 2010). Thus, we postulated that:

H5: Green product purchase attitude has a significant impact on consumers’ green product consumption behavioral intention.

### Mediating Effect of Attitude

Green product consumption attitude is a cognitive assessment of consumers whether the products are environmentally preserved (Lee, 2008; Sun et al., 2021). Dhir et al. (2021) identified the existing gap among environmental literacy, attitude, and behavior because environmental literacy influences an individual through environmental attitude. Chen and Chai (2010) believed that consumers perceived environmentally friendly products can influence consumers’ attitudes toward green product consumption behavioral intention. Many researchers pointed that consumers’ attitudes motivate consumption behavioral intention (Costell et al., 2010). Other previous literature investigated consumers’ perception and satisfaction as a significant component of consumers’ attitudes and loyalty (Costa et al., 2020; Rahman et al., 2021). Sadiq et al. (2021) reported that consumers’ attitude is a crucial dimension in determining consumption behavioral intention. The earlier studies reported that environmental attitude mediates the effect of environmental knowledge on consumption behavioral intention (Dhir et al., 2021). There is a positive association between green product purchase attitudes and behavioral intention (Sun et al., 2021; Zheng et al., 2021). In line with this, we postulate the following hypothesis:

H6: Green product purchase attitudes mediate the effect of (a) green product literacy, (b) green product orientation, and (c) social influence on green product consumption behavioral intention.

**FIGURE 1:**
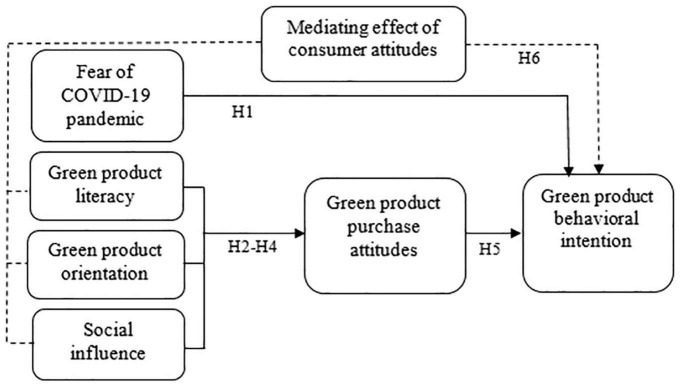
Conceptual model.

Based on the review of the literature, this work develops a conceptual model (Figure 1).

## Research Methodology

### Operationalization of Variables

Based on the review of literature, the variables of this work were adapted to the context of this study. The fear of COVID-19 pandemic was measured using three items adapted from Bitan et al. (2020), Mertens et al. (2020), and Sun et al. (2021). Green product literacy and green product orientation were evaluated using eight items adapted from Liobikienė and Poškus (2019). Three items were adapted based on Dhir et al. (2021) to evaluate the green product purchase attitude. Five items were modified from Nguyen et al. (2019) and Dhir et al. (2021) to evaluate the green-product consumption behavioral intention. For this work, we have used a five-point Likert scale (i.e., 1 = strongly disagree to 5 = strongly agree) to measure the respondent’s responses.

### Data Collection and Sample

The work employed an exploratory study, and data was collected from consumers in Malaysia. The respondents of this work were randomly selected from the consumer products, food and beverage shopping mall in the Kelang Valley area. This place has been chosen because it is the most densely populated and urbanized region, and many hypermarkets and supermarkets are located in this region. The research assistant of this work got permission from the manager of the shopping mall and physically distributed the questionnaires to the consumers. The consumers were politely asked to participate in the survey and ensured that the information would be kept confidential, and the exploratory results of this study would be used for academic purposes only. This work used the non-probability random sampling method. We randomly distributed the questionnaire to the respondents and requested them to submit the questionnaire to the information desk of the shopping mall. We distributed 1,000 questionnaires (between September and November 2020) to the respondents. We got the return of 512 responses and identified that nine responses were not completed. Thus, we considered 503 valid responses for data analysis with a response rate of 50.3%.

G*Power 3.1 version was used to check the robustness of the sample size and to assess the power of 503 samples. Faul et al. (2009) suggested that G*Power was used to ascertain the intensity of the sample. The conceptual model of this study shows five antecedents of sustainable green product consumption behavioral intention. Along these lines, G*Power with significant alpha (α) 0.05 provides the strength of 0.999, which is bigger than 0.80 (Chin, 2001). It implies that the acceptable degree of sample power of the current work is achieved, and the sample of 503 ensures the necessary capacity to dismiss null hypotheses (Faul et al., 2009).

## Data Analysis

### Demographic Information

The demographic findings reveal that male respondents (61.4%) were greater than female respondents (38.6%). The total sample of the study is classified into five different age groups: 18–24, 25–29, 30–34, 35–39, and 40–45, and the proportion of the age groups are 17.7, 66.2, 14.5, 0.8, and 0.8%, respectively. The majority of the respondents (66.2%) are married, followed by 25.4% who are single, and others. The majority (73.4%) of the respondents hold a bachelor’s degree. The respondents who have a master’s degree is 21.3%, while 0.2% of the respondents hold a doctoral degree. The respondents with a diploma and school certificate are 2.8 and 2.4%, respectively. The household income of the respondents is also categorized into 6 different income ranges. The highest portion of income lies in the range of RM 2001–RM 4000 (USD500–USD1000) whereas 24.3% of respondents earn an amount between RM 4001–RM 6000 (USD1001–USD1400). The ranges of RM 6001–RM 8000 (USD1401–USD1900), and RM 8001–RM10000 (USD1901–USD2000) are the incomes of 16.7 and 15.5%, respectively. The result reveals that 14.9% of the respondents earn below RM 2000 each month. The rest (0.2%) of respondents earn above RM 10000 (USD2000). The majority of the respondents (48.9%) purchase green products, which represents the highest number of buyers. Besides, 31.4% of respondents purchase toiletries and beauty green products, and the remaining purchase other products.

### Measurement Model Analysis

The study uses PLS statistical tool (SmartPLS 3.3) to evaluate the reliability and validity of the study using the measurement model test. The structural model was run for evaluating the hypotheses relationships among the constructs. PLS is a statistical algorithm for estimating causal links in a way that considers the modeling of interactions, correlated independents, error items, and measurement error. For this study, PLS analysis incorporates two levels of examination, such as the assessment of measurement model and structural model. Before examining the measurement model, the Harmon single-factor test was adopted to see whether there is any potential common method bias among the constructs (Podsakoff and Organ, 1986). Accordingly, the study uses the varimax rotation and the results revealed that eigenvalues are greater than 1.0. The factor estimated 41% of the variance, which is less than 50%, indicating that common method bias does not exist (Podsakoff et al., 2003).

The findings of measure model analysis indicate that all items loading exceeds the threshold value of 0.50, and Cronbach’s alpha (CA) values are greater than 0.70 for all constructs. The composite reliability (CR) value is larger than 0.70, and the average variance extracted (AVE) value exceed the cut-off point of 0.50 (Byrne, 2013; Hair et al., 2017). The threshold values of rho_A indicate greater than the recommended value of 0.70 (Table 1), which is suitable for composite reliability (Dijkstra and Henseler, 2015). Therefore, convergent validity is achieved in the study.

**TABLE 1:** Convergent validity.

Variables and items	FL	CA	rhoA	CR	AVE
**Fear of COVID-19 pandemic**		0.829	0.829	0.898	0.745
I am very worried about the effect of the COVID-19 pandemic for my health safety (fcp1).	0.868				
For my health, I find the virus to be much more dangerous than the seasonal flu (fcp2).	0.879				
My biggest coronavirus concern is about how long I will be able to handle isolation (fcp3).	0.842				
**Green product literacy**		0.716	0.721	0.840	0.637
I am very knowledgeable about green product that protects human health from various diseases (gpl1).	0.774				
I know how to select green products (gpl3).	0.804				
I understand the green symbols on product packages (gpl4).	0.815				
**Green product Orientation**		0.774	0.775	0.869	0.689
Human activities that exploit natural and biological resources endanger the environment (gpo1).	0.817				
I consider the environmental impact of my actions when making many of my consumption decisions (gpo2).	0.821				
I would describe myself as environmentally responsible (gpo3).	0.851				
**Social influence**		0.816	0.819	0.878	0.644
The purchase of eco-friendly products will make me a positive impression on other people (si1).	0.799				
The purchase of eco-friendly products will help me gain social approval (si2).	0.804				
Consumption of eco-friendly products will help me feel socially acceptable (si3).	0.792				
My choice of eco-friendly product is influenced by other consumers’ word of mouth (si4).	0.814				
**Green product purchase attitudes**		0.790	0.793	0.878	0.705
I think the green product consumption attitude will increase my healthy life (gpca1)	0.857				
I have favorable attitude toward purchasing and consuming a green product (gpca2)	0.864				
I prefer to buy green products that are harmless (gpca3)	0.796				
**Green product behavioral intention**		0.838	0.840	0.892	0.673
I purchase the environmental label and green food labeling products (spcb1).	0.800				
Given a choice between two substitute products, I intend to choose the one having less environmentally hazardous substances in the future (gpcb2).	0.837				
To me, it deserves to consume green products despite its premium pricing (gpcb3).	0.816				
I will purchase green products because they are less polluting (sgcb5).	0.828				

*FL, Factor Loading; CR, Cronbach’s Alpha; rhoA, Dillon-Goldstein’s rho; CR, Composite Reliability; AVE, Average Variance Extracted.*

Besides, for the robustness of the model fit, standardized root mean square residual (SRMR) is considered as a goodness-of-fit measure. The result reveals that the SRMR value of 0.05 indicates a satisfactory level for goodness-of-fit (Henseler and Sarstedt, 2013). The SRMR is an absolute measure of fit and is defined as the standardized difference between the observed correlation and the predicted correlation. A value less than 0.08 is considered a good fit (Pavlov et al., 2021). Henseler et al. (2016) reported SRMR as a goodness of fit measure for PLS-SEM that is used to avoid misspecification of the model.

Table 2 shows the discriminant validity and is evaluated based on the Fornell and Larcker (1981) criterion and Heterotrait and Monotrait (HTMT) ratio (Henseler et al., 2015). The findings show that the square root of AVE for each construct was higher than its correlation with another corresponding construct, which indicates that the constructs possess discriminant validity. For more robustness of the discriminant validity, we have considered the HTMT ratio of the correlation method. The result reveals that the discriminant validity does not violate the recommended HTMT value of 0.85 (Gold et al., 2001; Kline, 2011), which signifies that there is no multicollinearity issue between the items of the constructs.

**TABLE 2:** Discriminant validity.

	FCP	GPCB	GPL	GPO	GPPA	SI
**Fornell-Larcker criterion**						
FCP	0.863					
GPCB	0.518	0.820				
GPL	0.645	0.539	0.798			
GPO	0.610	0.544	0.638	0.830		
GPPA	0.556	0.626	0.590	0.618	0.840	
SI	0.612	0.605	0.676	0.714	0.702	0.802
**Heterotrait-Monotrait ratio (HTMT)**						
FCP						
GPCB	0.620					
GPL	0.839	0.691				
GPO	0.762	0.674	0.854			
GPPA	0.685	0.769	0.779	0.789		
SI	0.744	0.730	0.882	0.899	0.869	

*The square root of AVE for each construct presents on the diagonal. FCP, Fear of COVID-19 pandemic; GPL, Green product literacy; GPO, Green product orientation; SI, Social influence; GPPA, Green product purchase attitudes; GPCB, Green product consumption behavioral intention.*

### Structural Model Assessment

The collinearity statistics variance inflation factors (VIFs) are evaluated as an effective alternation capable to define the multicollinearity issue. Table 3 shows that the summarized results of the collinearity test and VIF scores for each construct are less than a threshold value of 3.3, indicating that the collinearity issue does not matter in this work (Diamantopoulos and Siguaw, 2006). The structural model indicates the causal relationship between independent and dependent constructs. According to Hair et al. (2017), the bootstrapping approach is used with a resampling of 5,000 for evaluating the significance of the path coefficient. The hypotheses relationship is shown in Table 3. The findings indicate that fear of COVID-19 pandemic (β = 0.246, *t* = 8.224, *p* < 0.01), green product literacy (β = 0.160, *t* = 5.179, *p* < 0.01), green product orientation (β = 0.188, *t* = 5.350, *p* < 0.01), and social influence (β = 0.459, *t* = 11.623, *p* < 0.01) are correlated with green product purchase attitude, respectively; thus, H1, H2, H3, and H4 are accepted. Similarly, green product purchase attitude has a significant impact on green consumption behavior (β = 0.490, *t* = 16.330, *p* < 0.01) and therefore H5 is also accepted.

**TABLE 3:** Path coefficients.

Hypothesis relationship		Beta	SD	*t*-value	VIF	*f* ^2^	*Q* ^2^	*R* ^2^	Decision
H1	FCP - > GPCB	0.246	0.030	8.224**	1.436	0.278			Accepted
H2	GPL - > FPPA	0.160	0.031	5.179**	2.022	0.127			Accepted
H3	GPO - > GPPA	0.188	0.035	5.350**	2.243	0.234			Accepted
H4	SI - > GPPA	0.459	0.040	11.623**	2.449	0.185	0.384	0.533	Accepted
H5	GPPA - > GPCB	0.490	0.030	16.330**	1.446	0.293	0.236	0.434	Accepted
**Mediating effect of attitude**									
H6a	GPL - > GPPA - > GPCB	0.078	0.016	4.776**					Accepted
H6b	GPO - > GPPA - > GPCB	0.225	0.024	9.193**					Accepted
H6c	SI - > GPPA - > GPCB	0.225	0.024	9.193**					Accepted

FCP, Fear of COVID-19 pandemic; GPL, Green product literacy; GPO, Green product orientation; SI, Social influence; GPPA, Green product purchase attitude; GPCB, Green product consumption behavioral intention; VIF, Collinearity Statistics. t-value ≥ 2.326 considers **p < 0.01.

It is also evident that green product purchase attitude mediates the effect of green product literacy (β = 0.078, *t* = 4.776, *p* < 0.01), green product orientation (β = 0.225, *t* = 9.193, *p* < 0.01), and social influence (β = 0.225, *t* = 9.193, *p* < 0.01) on green consumption behavior; thus, H6a, H6b, and H6c are supported. Cohen (1988) reported that it is important to examine the effect sizes (*f*^2^) of the indicators and that *f*^2^ values of 0.35, 0.15, and 0.02 are considered high, moderate, and small effect sizes, respectively (Table 3). Hence, the effective size was found moderate for all the constructs. The findings of R-square value of 53.3% for green product purchase attitude is explained by green product literacy, green product orientation, and social influence, whereas 43.4% of green product consumption behavioral intention is explained by the fear of COVID-19 pandemic and consumers’ green product purchase attitude. It suggests that each construct possesses enough capability to explain green product consumption behavioral intention. Following Stone (1974) and Geisser (1975), the Q-square is examined using the blindfolding procedure to evaluate the predictive relevance of the model. Hence, the findings indicate that Q-square values of consumers’ green product purchase attitude (0.384) and green product consumption behavioral intention (0.236) are larger than zero, indicating the predictive relevance of the model. Fornell and Cha (1994) suggested that a Q-square value greater than zero specify acceptable predictive capability for the model.

### Importance Performance Matrix

The importance-performance matrix (IPM) is adopted to evaluate the robustness of the results. Ringle and Sarstedt (2016) stated that IPM emphasizes the factors to develop a certain target factor. The results indicate that IPM achieves the total effects of the relationship with constructs of COVID-19 pandemic, green product literacy, green product orientation, social influence, and attitude on the target construct of green product consumption behavioral intention to identify their importance. Table 4 shows that consumers’ fear of COVID-19 pandemic is an important factor in targeting green consumption behavioral intention since it is reflected by high importance and performance value, which is followed by, green product literacy, green product orientation, social influence, and green product purchase attitude.

**TABLE 4:** Performance and total effects.

Target factor: green product consumption behavioral intention	Total effects	Performances
Fear of COVID-19 pandemic	0.246	77.384
Green product literacy	0.078	74.446
Green product orientation	0.092	73.405
Social influence	0.225	71.998
Green product purchase attitude	0.490	70.974

## Discussion

Green product consumption behavioral intention has become popular, and green marketing has captured the attention of practitioners and academic scholars. This study explores the predictors of green product consumption behavioral intention during the COVID-19 pandemic. The findings revealed that the fear of COVID-19 pandemic reflects consumers’ green product consumption behavioral intention. This finding is related to Sun et al. (2021), who discussed the green consumption behavioral intention and consumers’ positive and negative impact of COVID-19 pandemic in the context of China. Panjaitan and Sutapa (2010) point out that green product is an ecological product in the process that has a great impact on human health.

The findings of this work also identified the dominant effect that green product literacy/knowledge exerts on consumers’ attitudes. This finding appears to ensure the results of earlier studies (Pagiaslis and Krontalis, 2014; Yadav and Pathak, 2016). The finding implies that green product literacy leads a consumer to higher levels of involvement with green product purchase attitude, which in turn leads to green product behavioral intention. Green product literacy can reflect consumers’ attitude to purchase a green product that reflects the healthiness of people (Sun et al., 2021). The results revealed that green product orientation has a significant impact on consumers’ green product purchase attitudes. This result is related to Fraj-Andrés et al. (2009) and Zhou et al. (2020) who emphasized green product orientation for the purchasing involvement of people and the impact social influence exerts on green product consumption attitude. This is following the results of Xie et al. (2019) and Dhir et al. (2021) explained the social influence factors and attitude.

The results identified that consumers’ green product purchase attitudes have a significant influence on behavioral intention. This finding is related to a previous work (Dhir et al., 2021). Consumers’ attitudes can contribute to the transformation toward a sustainable society. A green product purchase attitude will increase demand customers toward green consumption behavioral intention. The result also demonstrated that consumers’ attitude is recognized as one of the key influence factors to achieve green consumption behavioral intention. This finding is supported by Sun et al. (2021) who focused on the consumers’ attitude in sustainable green consumption behavioral intention. The findings of this study imply that due to the uncertainty of COVID-19 pandemic, many consumers are purchasing green products for their good health. Green product is an eco-friendly product and sustainable product designed to minimize environmental impacts during the whole life-cycle that is not harmful to health.

## Conclusion

### Theoretical and Managerial Implications

This study contributes to customers’ green consumption behavioral intention along with fear of COVID-19 pandemic, green product literacy, green product orientation, social influence, and consumers’ green product attitude that lead to consumers’ green consumption behavioral intention. The findings of this study will provide valuable insights for policymakers and marketing managers about the key determinants that influence sustainable green consumption behavioral intention. The marketing operators can take the initiative to promote ecofriendly products to consumers. The results reveal that fear of COVID-19 pandemic, green product orientation, and green product literacy are the crucial factors that reflect consumer purchase attitude toward green consumption behavioral intention. Policymakers and managers should understand customers’ perceptions and knowledge about the green product consumption behavioral intention. The fear of COVID-19 pandemic can influence consumers from enduring commitment to social communities and natural resources. This study also uses the theory of fear to explain consumers’ green product consumption behavioral intention. The results indicated that green product literacy, green product orientation, fear of COVID-19 epidemic, and social influence can promote green product consumption behavioral intention.

The consumers’ green product purchase attitudes due to the fear of the COVID-19 pandemic can increase consumers’ green product consumption behavioral intention. It implies that a higher green product purchase attitude may increase higher green product consumption behavioral intention. Marketers should highlight convincing the customers’ attitudes, which may be a suitable marketing strategy for attracting people toward green product consumption behavioral intention. Marketers should not only focus on product characteristics but also an emphasis on the consumers’ attitudes toward their green product consumption behavioral intention. It is difficult for some consumers to identify green products; thus, green product literacy or knowledge is necessary to identify the green product. The manufacturing companies should introduce an ecofriendly product that reflects consumers’ green product consumption behavioral intention. The government can monitor the ecolabels of green products and processes to ensure customer attitudes toward sustainable green product consumption behavioral intention.

### Limitation and Future Research

The limitation of this study can be generalized in that the data was collected at a single point in time, and the results are reported based on the data analysis. Therefore, the findings might be reflected by the fact that this work cannot perceive the dynamic changes of the consumers green product attitudes toward behavioral intention of green products. Besides, this study is cross-sectional; the future work can use a longitudinal tactic to afford findings that are more precise. The sample population was limited to Malaysian citizens, which may restrict the generalizability of the outcomes. Future investigations can explore the existing model of this study with other nations. Moreover, this study highlighted the consumers’ attitudes toward ecofriendly or green product and their impact on their green product consumption behavioral intention. However, personal protective equipment can influence consumers’ purchase and behavioral intention of green products during and the ongoing uncertainty of COVID-19 pandemic. Thus, the future work can be conducted to test with other social and behavioral factors such as personal protective equipment and social sustainability for green product consumption behavioral intention.

## Data Availability Statement

The original contributions presented in the study are included in the article/supplementary material, further inquiries can be directed to the corresponding author/s.

## Author Contributions

XC and MKR designed the overall framework of the research and drafted the manuscript. MSR and MG conducted a literature review. MAR collected the data and reviewed the draft. NCN revised the manuscript and reviewed the draft. All authors read the final manuscript and approved it for final submission.

## Conflict of Interest

The authors declare that the research was conducted in the absence of any commercial or financial relationships that could be construed as a potential conflict of interest.

## Publisher’s Note

All claims expressed in this article are solely those of the authors and do not necessarily represent those of their affiliated organizations, or those of the publisher, the editors and the reviewers. Any product that may be evaluated in this article, or claim that may be made by its manufacturer, is not guaranteed or endorsed by the publisher.
